# Syllables are Retrieved before Segments in the Spoken Production of Mandarin Chinese: An ERP Study

**DOI:** 10.1038/s41598-019-48033-3

**Published:** 2019-08-13

**Authors:** Chen Feng, Yuan Yue, Qingfang Zhang

**Affiliations:** 1 Department of Psychology, Renmin University of China, Beijing, 100872 China; 2 Key Laboratory of Behavioral Science, Institute of Psychology, Chinese Academy of Sciences, Beijing, 100101 China

**Keywords:** Language, Human behaviour

## Abstract

Languages may differ in terms of the functional units of word-form encoding used in spoken word production. It is widely accepted that segments are the primary units used in Indo-European languages. However, it is controversial what the functional units (syllables or segments) in Chinese spoken word production are. In the present study, Mandarin Chinese speakers named pictures while ignoring distractor words presented simultaneously, which shared atonal syllables, bodies or rhymes, or were unrelated with the name of the target pictures. Behavioral results showed that naming latencies in the 3 phonologically-related conditions were significantly shorter than those associated with the unrelated condition. EEG data indicated that the syllable-related condition modulated event-related potentials (ERPs) in a time window of 320–500 ms, the body-related condition modulated ERPs from 370–420 ms, while the rhyme-related condition modulated ERPs from 400–450 ms. The starting points for evident syllable, body, and rhyme priming effects were 322 ms, 368 ms, and 408 ms (by the Guthrie & Buchwald method) or 340 ms, 372 ms and 403 ms (by the jackknife procedure), respectively. Our findings provide a relative temporal course of syllable and segment encoding in Chinese spoken naming: Syllables are retrieved before segments, and constitute the primary processing units during the early stage of word-form encoding. Furthermore, segments and their order are retrieved incrementally from left to right when producing Chinese spoken words.

## Introduction

Speech production involves conceptual preparation, lexical selection, word-form encoding, and articulation processes. In the model of language production proposed by Levelt and colleagues^1^, word-form encoding consists of segmental and metrical retrievals, both of which are then combined into a syllable via a syllabification process. The role of sub-lexical units such as the phoneme, syllable, and mora in this process has received much attention^2–7^.

Evidence for phonemes as the primary units in spoken word production comes from various Indo-European languages, such as Dutch^8–11^, English^6,12–14^, Russian^15,16^, and Persian^17^, with different tasks including the implicit priming paradigm, masked priming paradigm, and phoneme repetition. For example, in a masked-form priming paradigm, Dutch participants named bisyllabic words which were preceded by visually masked primes. When primes shared the initial segment(s) with the target, naming latencies were significantly shorter than when the segments were not shared. Damian and Dumay^12^ reported that when participants produced adjective-noun phrases in response to colored objects, a single-phoneme overlap between the color and object name facilitated responses (e.g., red rope), even when the overlapping phoneme occupied different positions within the words^14^ (e.g., green flag). These findings support the view that phonemes are the primary functional units of word-form encoding.

The claim that phonemes are the primary phonological encoding units in Indo-European languages is plausible because the orthographies of these alphabetic languages explicitly code for phonemes via letters or letter combinations. However, what about non-alphabetic languages such as Chinese? Mandarin Chinese has approximately 400 atonal syllables, all of which have a very straightforward structure, and no resyllabification is required for speaking. This variation in phonological properties suggests that it cannot be directly assumed that phonemes are equally important across all languages, therefore making it necessary to investigate the specific encoding mechanisms of different languages.

Recently emerging evidence offers some support for the possibility that for Chinese speakers, syllables rather than phonemes constitute the primary units of word-form encoding. This was supported by earlier behavioral findings of a reliable syllabic priming effect but a phoneme priming null effect during word production of Mandarin^2,3,18–23^. The role of briefly-presented prime words which matched or mismatched their first syllable structures with spoken Chinese words has been examined in a masked priming task^2^. They found that CV targets (C represents consonants and V represents vowels) were named faster when preceded by CV primes compared to CVG primes (G represents a glide sound), while the opposite pattern was discovered for CVG targets. Similar results were reported in both word reading and picture naming^20^. Most recently, the syllabic priming effect has been replicated, with little evidence of phonemic priming^22^; in fact, phonemically-related primes elicited a slightly inhibitive effect when compared to phonemically-unrelated primes. In a delayed picture-naming study using electrophysiological measurements^21^, participants were required to name pictures upon a response cue. Each set of two consecutively presented pictures formed a pair consisting of prime and target, which either shared the same word-initial syllable or phoneme or were unrelated. The shared-syllable condition elicited an effect in the time windows of 200–400 ms and 400–600 ms, while no significant event-related potentials (ERP) effects were observed for the shared-phoneme condition.

A few recent studies, however, using other linguistic tasks, have reported sub-syllabic priming effects in Chinese spoken word production. Using a picture-word interference task, significant facilitation effects were found when the target and the distractor word shared syllables, or when only the final segments were shared^24^, reflecting the fact that a sub-syllabic unit might be a possible candidate for a functional unit. Furthermore, studies using electrophysiological measurements found differences between shared and unshared phoneme conditions^25,26^. Using a phoneme repetition task, Qu *et al*. reported that phoneme repetition modulated ERPs 200 ms post-target picture onset when the color and object name shared the initial phoneme^25^. A similar finding^26^ was observed for an implicit priming paradigm which showed that phoneme repetition evoked more positive ERPs in the time window of 180–300 ms post-target picture onset. Also, in a functional magnetic resonance imaging (fMRI) study^27^, distinctive brain regions were found to exhibit neural adaptation effects for phonemes (bilateral basal ganglia) and syllables (bilateral superior temporal gyrus). Taken together, these results suggest that phonemes constitute functional units of phonological encoding even in Chinese spoken word production.

Although these findings paint a complex picture, a plausible scenario is that speakers of all languages mentally represent phonemes and syllables, but attribute differential importance and temporal courses depending on the target language. To explain these various findings, O’Seaghdha *et al*. proposed a proximate unit principle^3,28^ which assumes that phonological units below the word vary across languages. This principle has been integrated into the influential spoken production model^29^, WEAVER++, which assumes that in its original form, phonological encoding in languages involves a parallel activation of phonemic segments and metrical frames in the spoken production system, followed by prosodification, which concerns serial content-to-frame association and syllabification. For Chinese speakers^3^, atonal syllables are associated with the tonal frame serially as the first selectable units below the lexeme. This principle has also been validated by Roelofs^29^ in a computer simulation model. Furthermore, in order to explain the null onset effect in Chinese spoken word production, Roelofs^29^ assumed a parallel selection of phonemes within a syllable. By contrast, Chen *et al*. proposed the opposite assumption that segments are selected in sequence from initial to final phonemes^22^. In a picture-word interference (PWI) task with manipulation of stimulus onset asynchrony (SOA) between pictures and distractor onset, Wang, Wong, and Chen^30^ found a facilitation effect of shared atonal syllables when distractors were presented 100 ms before (SOA = −100 ms) or simultaneously with (SOA = 0 ms) the target picture, and a facilitation effect of shared body segments (includes the onset consonant and the vowel with targets) that only appeared at 0 ms SOA. The effects of shared rhyme segments (includes the vowel and the final consonant with targets) were absent in both SOAs, suggesting a serial processing order for segment retrieval in Chinese spoken word production. However, a behavioral study cannot provide fine-grained temporal courses of body and rhyme segments encoding.

A critical factor distinguishing these versions is the time course of different phonological units. For Chinese spoken word production, the retrieval of syllables precedes phonemes, and phonemes within a syllable are processed simultaneously according to a parallel mechanism^29^, but sequentially according to a serial mechanism^22^. Investigating these phenomena requires a technique involving high-resolution time measurements, such as EEG. Although there has been evidence from ERP studies indicating the involvement of segments at a quite early stage^25,26^ (i.e., around 200 ms after picture onset), Wong *et al*. argued that this early segmental processing might arise from facilitation or competition between similar syllable nodes in the syllables retrieval stage because shared segments increased similarities among syllables^21^. Previous EEG studies in Chinese examined the time course of syllable retrieval and phoneme retrieval separately^21,25,26^. It is risky to infer the interval between syllabic and phonemic encoding in spoken word production via comparisons among studies due to the different tasks and different participants involved in those studies^31,32^. As a consequence, it is critical to include a shared-syllable condition as well as shared-phoneme/segment-condition in an experiment for identical participants. In a delayed-prime picture-naming study reported by Wang and colleagues^21^, each 2 consecutive pictures implicitly formed a pair of prime and target, whose names shared either the same atonal syllable or the same initial segment, or were unrelated in the control condition. Participants were asked to first prepare and later produce disyllabic Chinese words upon picture prompts when a response cue was presented. These researchers found a widely distributed positivity in the 200- to 400-ms interval and an anterior positivity in the 400- to 600-ms interval for the shared-syllable condition, and a null effect for the initial-segment shared condition.

There are two possible interpretations of the null finding for the initial-segment effects. First, there was only one phoneme shared between primes and targets in the initial-segment shared condition. Therefore, the activation of single phonemes may have been too weak to be detectable. Second, the pictures as primes in combination with a delayed naming task might be insensitive to the phonemic activation of distractor pictures. In fact, using a typical picture-word interference (PWI) paradigm, an effect from the initial-segment or body shared condition on naming latencies was observed consistently in several studies^30,33^. Using a masked priming task, Zhang and Damian recently reported a syllabic overlap effect in the 300- to 400-ms interval, as well as a phoneme overlap effect in the 500- to 600-ms interval after picture onset in Mandarin spoken word production^34^. However, given the interval (around 100 ms) between the syllabic overlap effect and phoneme overlap effect, the phoneme overlap effect in this study may have arisen as the result of phonetic encoding or self-monitoring. Thus far, findings concerning phoneme overlap have been mixed and inconclusive.

In this article, we address the limitations of previous studies. We used a typical picture-word interference task, and included an atonal syllable-related condition, a body-related condition sharing the first two phonemes (including the onset consonant and the vowel) with targets, a rhyme-related condition sharing the final two phonemes (including the vowel and the final consonant) with targets, and an unrelated condition between distractors and targets. We recorded both the behavioral and electroencephalography (EEG) signals concurrently. Data from each distractor type were then compared with the unrelated condition. According to the proximate unit principle, we expected an early syllable priming effect and a later body or rhyme priming effect. Specifically, with regard to the temporal courses of the body-related and rhyme-related conditions, we expected a parallel time course according to Roelofs’s assumption^29^, but a serial time course according to Chen *et al*.’s assumption^22^.

## Results

### Behavioral results

One participant was excluded from the analysis due to large electrode drift and excessive artefacts. In addition, incorrectly named trials (1.4%), as well as latencies shorter than 500 ms, longer than 1,500 ms (2.5%), or beyond 3 SDs (1.7%) were excluded from the analysis. The mean naming latencies were 708 ms (SE = 4.19 ms) for the syllable-related condition, 730 ms (SE = 4.45 ms) for the body-related condition, 733 ms (SE = 4.61 ms) for the rhyme-related condition, and 746 ms (SE = 4.57 ms) for the unrelated condition.

The data were analyzed using a linear mixed-effects model (LMM)^35,36^. The LMM includes random effects on the subject level and the item level simultaneously. Consequently, a linear mixed-effects model contains a fixed effect part, a random effect part, and residuals. For example, the model of the omnibus estimation for latency was:

$$\begin{array}{ccc}{\rm{L}}{\rm{a}}{\rm{t}}{\rm{e}}{\rm{n}}{\rm{c}}{\rm{y}} & \sim  & ({\rm{d}}{\rm{i}}{\rm{s}}{\rm{t}}{\rm{r}}{\rm{a}}{\rm{c}}{\rm{t}}{\rm{o}}{\rm{r}}\,{\rm{t}}{\rm{y}}{\rm{p}}{\rm{e}}\ast {\rm{b}}{\rm{l}}{\rm{o}}{\rm{c}}{\rm{k}})+(1+{\rm{d}}{\rm{i}}{\rm{s}}{\rm{t}}{\rm{r}}{\rm{a}}{\rm{c}}{\rm{t}}{\rm{o}}{\rm{r}}\,{\rm{t}}{\rm{y}}{\rm{p}}{\rm{e}}+{\rm{b}}{\rm{l}}{\rm{o}}{\rm{c}}{\rm{k}}|{\rm{s}}{\rm{u}}{\rm{b}}{\rm{j}}{\rm{e}}{\rm{c}}{\rm{t}})\\  &  & +(1+{\rm{d}}{\rm{i}}{\rm{s}}{\rm{t}}{\rm{r}}{\rm{a}}{\rm{c}}{\rm{t}}{\rm{o}}{\rm{r}}\,{\rm{t}}{\rm{y}}{\rm{p}}{\rm{e}}+{\rm{b}}{\rm{l}}{\rm{o}}{\rm{c}}{\rm{k}}|{\rm{i}}{\rm{t}}{\rm{e}}{\rm{m}})\end{array}$$

In the equation we used, (distractor type * block) represents the fixed effects (the main effects of distractor type and block, as well as their interaction), (1 + distractor type + block|subject) indicates that each subject has a distinct intercept and slope, and (1 + distractor type + block|item) indicates that each item has a unique intercept and slope. According to the guidelines proposed by Barr, Levy, Scheepers, and Tilfor^37^, the maximal random effects structure was constructed in order to obtain a more reliable estimation. The lmer function of the lme4 package was used to estimate the fixed effects and parameter estimation of the LMM. The degrees of freedom and *p*-values were computed using the ANOVA function of the lmerTest package with the Satterthwaite approximations. These analyses were conducted using R free software^38^.

For naming latencies, the main effect of distractor type was significant, *F*_(3, 21)_ = 14.59, *p* < 0.001, as was the main effect of block (*F*_(2, 19)_ = 8.64, *p* < 0.01), whereas the interaction between distractor type and block was not significant (*F*_(6, 3,732)_ = 1.42, *p* = 0.20). Multiple comparisons (*p* values with FDR correction) were then conducted for each phonologically-related condition. All 3 phonologically-related conditions were named significantly faster than the unrelated condition (syllable-related: *t*_(18)_ = 6.44, *p*_adjusted_ < 0.001, Cohen’s *d* = 0.983; body-related: *t*_(17)_ = 3.1, *p*_adjusted_ < 0.01, Cohen’s *d* = 0.465; rhyme-related: *t*_(17)_ = 2.13, *p*_adjusted_ < 0.05, Cohen’s *d* = 0.456).

### Electrophysiological results

Time windows were selected based on the results of an analysis of consecutive 10-ms time windows. First, the 3 phonologically-related conditions were compared in each region of interest (ROI) with the unrelated condition via serial paired *t*-tests with a step size of 10 ms. The time windows were selected when at least 5 consecutive *t*-tests approached significance (*p* < 0.05, two-tailed) for each related condition at one ROI.

Three time windows were selected (320–500 ms post-picture onset for the syllable-related condition, 370–420 ms for the body-related condition, and 400–450 ms for the rhyme-related condition). In each time window, an omnibus ANOVA was conducted for distractor type (syllable-related, body-related, or rhyme-related vs. unrelated) and ROI. For the syllable-related and unrelated comparison in the time window of 320–500 ms, the main effects of distractor type and ROI were significant (distractor type: *F*_(1, 18)_ = 4.78, *p* < 0.05; ROI: *F*_(8, 144)_ = 10.43, *p* < 0.001), and the interaction between distractor type and ROI was also significant (*F*_(8, 144)_ = 3.88, *p* < 0.001). For the body-related and unrelated comparison in the time window of 370–420 ms, the main effects of distractor type and ROI were significant (distractor type: *F*_(1, 18)_ = 4.67, *p* < 0.05; ROI: *F*_(8,144)_ = 11.45, *p* < 0.001), and the interaction between distractor type and ROI was not significant (*F*_(144, 8)_ = 1.71, *p* = 0.1). For the rhyme-related and unrelated comparison in the time window of 400–450 ms, the main effect of distractor type was not significant, the main effect of ROI was significant (distractor type: *F*_(18,1)_ = 2.3, *p* = 0.146; ROI: *F*_(144,8)_ = 13.89, *p* < 0.001), and the interaction between distractor type and ROI was only marginally significant (*F*_(144,8)_ = 1.87, *p* = 0.069).

**Figure 1:**
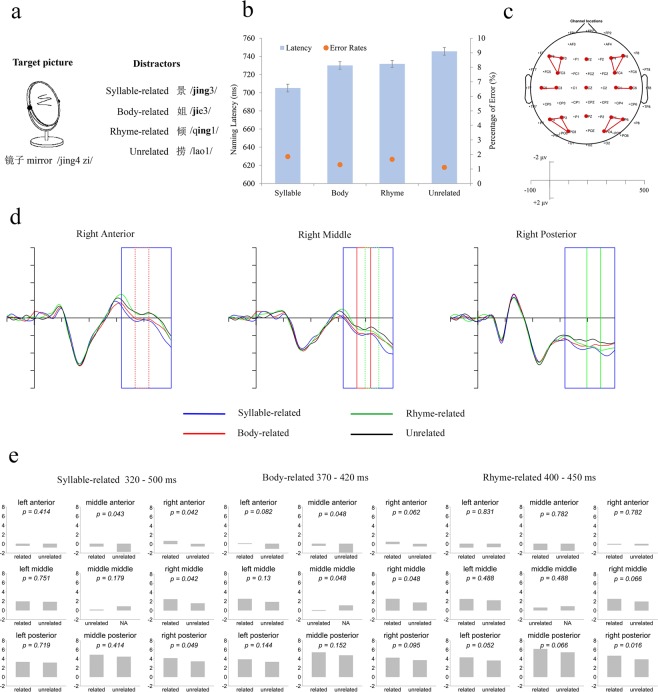
(**a**) Example of stimuli used in this study with a target picture and its four corresponding distractors; (**b**) Average latencies for each condition. All three phonologically-related conditions had shorter latencies than the unrelated condition. Error bars represent standard errors. (**c**) Regions of interest (ROI) and axis scale; (**d**) Grand average ERPs for the four phonologically-related/unrelated conditions (blue: syllable-related; red: body-related; green: rhyme-related; black: unrelated) in 3 ROIs: right-anterior (F4, F6, FC4), right-middle (C4, C6), and right-posterior (P4, P6, PO4); Rectangles with colors corresponding to conditions are used to mark significance (solid lines) or marginal significance (dashed lines). (**e**) Results of paired *t*-test for each comparison in corresponding time windows. All *p* values were corrected with the FDR method.

Furthermore, each of the three phonologically-related conditions was compared to the unrelated condition in each ROI, and FDR correction was applied in order to avoid the multiple comparison problem. For the syllable-related and unrelated comparison in the time window of 320–500 ms, four ROIs showed significant differences: the middle anterior (*t*_(18)_ = 2.71, *p*_adjusted_ = 0.043, Cohen’s *d* = 0.62), right anterior (*t*_(18)_ = 3.11, *p*_adjusted_ = 0.042, Cohen’s *d* = 0.71), right middle (*t*_(18)_ = 2.91, *p*_adjusted_ = 0.042, Cohen’s *d* = 0.67) and right posterior (*t*_(18)_ = 2.51, *p*_adjusted_ = 0.049, Cohen’s *d* = 0.57). For the body-related and unrelated comparison in the time window of 370–420 ms, the results showed significant (or marginally significant) differences in the left anterior (*t*_(18)_ = 2.15, *p*_adjusted_ = 0.082, Cohen’s *d* = 0.49), middle anterior (*t*_(18)_ = 2.66, *p*_adjusted_ = 0.048, Cohen’s *d* = 0.61), right anterior (*t*_(18)_ = 2.39, *p*_adjusted_ = 0.062, Cohen’s *d* = 0.55), and middle central (*t*_(18)_ = 2.69, *p*_adjusted_ = 0.048, Cohen’s *d* = 0.62). For the rhyme-related and unrelated comparison in the time window of 400–450 ms, four ROIs showed significant (or marginally significant) differences: the right middle (*t*_(18)_ = 2.37, *p*_adjusted_ = 0.066, Cohen’s *d* = 0.54), left posterior (*t*_(18)_ = 2.81, *p*_adjusted_ = 0.052, Cohen’s *d* = 0.64), middle posterior (*t*_(18)_ = 2.42, *p*_adjusted_ = 0.066, Cohen’s *d* = 0.56), and right posterior (*t*_(18)_ = 3.67, *p*_adjusted_ = 0.016, Cohen’s *d* = 0.84). The averaged ERP waveforms of three typical ROIs as well as the detailed results of the paired *t*-test for each comparison in each ROI are displayed in Fig. 1d,e.

To identify time points at which the ERPs between two conditions (related versus unrelated) began to diverge, we adopted two methods to identify the starting point of the observed effects: the Guthrie & Buchwald method^39^ and the jackknife-based procedure^40^. The Guthrie & Buchwald method was developed by Guthrie and Buchwald^39^ and was designed to prevent erroneous detections of short significant intervals through computer simulation. An interval would be declared to be significant if its observed number of consecutive significant time points was larger than the estimated time interval, where the initial time point would be taken as the starting point of an effect. Based on the results of the mean amplitude analyses, electrode Fz was selected as the representative electrode for the syllable-related condition and the body-related condition, while P4 was selected to represent the rhyme-related condition. Table 1 lists the results of this analysis for different phonologically-related conditions versus the unrelated condition. For the syllable-related condition, the starting point was 322 ms post-picture onset and the effect lasted 178 ms; for the body-related condition, the starting point was 368 ms and the effect lasted 96 ms; for the rhyme-related condition, the starting point was 408 ms and the effect lasted 52 ms. The jackknife-based procedure, unlike the Guthrie & Buchwald method, defined the starting point of an effect as the time point at which the amplitude of the difference waveform reached 50% of the peak amplitude. This procedure runs N calculations for an ERP study which has N subjects. In each calculation, one of the N subjects’ difference wave is to be excluded, and one starting point will be calculated on the averaged difference waveform of the remained N-1 subjects. The mean value of the N starting points is to be regarded as the starting point of an effect. Results indicated that the syllable effect (340 ms) was earlier than the body effect (372 ms), and the body effect was earlier than the rhyme effect (403 ms). Therefore, both methods consistently showed an early syllable effect and a later segments effect in speaking.

**Table 1:** Results of starting point analysis by Guthrie & Buchwald test for each comparison.

Comparison	Suggested minimum consecutive time points	Consecutively significant time points (max)	Starting point (ms)
Syllable-related *vs*. Unrelated	14	89	322
Body-related *vs*. Unrelated	16	48	368
Rhyme-related *vs*. Unrelated	12	26	408

## Discussion

The current study investigated the time courses of syllabic and segmental encoding during overt spoken word production via a PWI paradigm. The naming latencies of all three phonologically-related conditions were significantly shorter than that of the unrelated condition, reflecting the involvement of syllabic and sub-syllabic units (such as bodies and rhymes) in spoken word production. More importantly, ERP data provided a detailed depiction of the time courses for processing different units: a syllable-related effect from 320–500 ms in the middle anterior, right anterior, right middle, and right posterior; a body-related effect from 370–420 ms mainly in the anterior area; and a rhyme-related effect from 400–450 ms in the left posterior, middle posterior, right posterior, and right middle area. The starting point analysis by the Guthrie & Buchwald method showed that the initiation of effects began at 322 ms for syllable-related effects, 368 ms for body-related effects, and 408 ms for rhyme-related effects (or 340 ms, 372 ms, 403 ms respectively by the jackknife procedure), suggesting a 46/32-ms interval from syllable retrieval to body retrieval and a 40/31-ms interval from body retrieval to rhyme retrieval.

The time intervals we found for syllable priming (320–500 ms), body priming (370–420 ms), and rhyme priming (400–450 ms) are roughly in agreement with the time course of phonological encoding (275–455 ms) introduced by Indefrey and Levelt^41,42^. Mean latencies in our study were about 730 ms, which is somewhat slower than the 600 ms estimate from the meta-analysis by Indefrey and Levelt^41,42^. The temporal course of syllabic and segmental encoding is broadly in line with the results of studies in alphabetic languages and Mandarin Chinese using ERP methodology. For example, Wang *et al*. found a syllable priming effect in the 200–400 ms interval in Chinese spoken word production using a delayed naming task^21^. Zhang and Damian found a syllable effect in the time window of 300–400 ms and a phoneme effect from 500–600 ms using a masked priming paradigm^34^. These results were interpreted as implying that syllables are the proximate units of phonological encoding. With regard to location, syllable priming effects presented in the right anterior and posterior areas, body priming effects presented in the left and right anterior areas, while rhyme priming effects presented in the left and right posterior areas, suggesting that distinct EEG patterns are involved for encoding syllables, bodies, and rhymes. Specifically, all phonologically-related conditions were found to elicit less-negative waveforms than the unrelated condition, which is consistent with previous findings about phonological effects^21,26,43–45^.

Critically, we first observed that the syllabic effect was 46/32 ms earlier than the body effect. According to the WEAVER++ model incorporated with the proximate units principle, syllabic “chunks” are retrieved at an early stage of word-form encoding, followed by the retrieval of syllable segments. The 46/32 ms interval indicated that syllables are retrieved before segments in word-form encoding. There are two possible temporal patterns for this time interval. One is that Chinese speakers retrieve syllables as proximate units firstly, and then dividing it into phonemes or segments for articulation preparation, resulting in a serial pattern. For this temporal pattern, we suggest that speakers take approximately 40 ms to retrieve a syllable (including three phonemes in average) in speaking, which was in line with previous findings in English^46,47^. Wheeldon and Levelt found that in phoneme monitoring, the reaction time difference between the onset and the offset of the first CVC syllable (three phonemes) was 55 ms^46^. Van Turennout *et al*. observed a slightly larger 80-ms difference between monitoring a word’s onset and its offset in a dual-choice go/no-go task^47^, although they used targets that were 50% longer than those of Wheeldon and Levelt^46^. An alternative explanation is that segment retrieval begins before syllable retrieval has been completed, which may result in a cascaded pattern for syllabic and segmental encoding in the production of Chinese words. These findings suggest that atonal syllables are the primary functional units of phonological encoding below the word level in Chinese spoken word production, thus providing strong evidence for the proximate units principle.

However, the starting points in this study showed that bodies were retrieved 40/31 ms before rhymes, which is contradicted by the parallel processing proposition^3,29^. Models of spoken word production assume that a morpheme’s segments or phonemes become simultaneously available, but with labeled links indicating their correct ordering^48^. Furthermore, Indefrey and Levelt proposed that 20 ms of encoding time per phoneme is required during the syllabification process in alphabetic languages^41,42^. Thus, a 40/31 ms time interval between body and rhyme fits with their estimation. We therefore suggest that Chinese speakers process the content of segments and their order after syllable retrieval, and spelled-out segments are successively inserted into the present metrical template. The sequential processing order of bodies and rhymes in the present study may reflect a combination of segments and their positions at a later stage of word-form encoding^48^. During the segments encoding, speakers divide a syllable into segments, and they treat the body and the rhyme as components of a syllable equally. However, note that a body prime can constitute a whole syllable while a rhyme prime cannot, which might influence the temporal courses of the body and the rhyme. We also suggest that it is necessary to replicate the finding concerning the time interval between body-related and rhyme-related segments in a future independent study.

It should be noted that this interpretation is not in line with the findings of previous researchers. Yu, Mo, and Mo found that both initial and non-initial phonemes modulate ERPs in the 180–300-ms interval, and suggest a parallel explanation^26^. However, the time interval they selected began at 180 ms, which is too early for the word-form encoding stage in spoken word production. Meanwhile, the *p* values in Yu *et al*.’s study^26^ were not corrected for multiple comparison (see also Qu, Damian, and Kazanina^25^ for a similar problem). We examined their *p* values and discovered that not all *p* values were significant according to the FDR correction method. This problem was avoided in the present study.

The current study found reliable syllabic priming effects and sub-syllabic segmental priming effects simultaneously on naming latencies using a typical picture-word interference paradigm^24,30,49^. In contrast, previous studies employing various tasks consistently found a syllable-shared effect but no phoneme-overlap effect. For example, using a form preparation (or implicit priming paradigm), J.-Y. Chen *et al*. reported a syllable benefit but no first-consonant preparation benefit^18^ (see also Chen and Chen^23^). Using a masked priming task, J.-Y. Chen *et al*. reported a facilitation effect in the shared-syllable condition^22^. Interestingly, they also found a weakly negative effect in the shared-initial consonants condition^22^ (see also Zhang and Damian^34^).

Concerning the polarity of the phoneme overlap effect, it was facilitative in the picture-word interference task^23,24,33,50^, whereas it was weakly inhibitive in the masked priming task^22^. The distractors were presented briefly and then masked by a backward mask in the masked priming task. Due to visible and invisible distractors in the typical and masked PWI tasks^51^, it is possible that the phonological relationships were more obvious in the PWI task than in the masked priming task. Using the PWI task, Yue and Zhang found a segmental facilitation effect in immediate naming, whereas a segmental inhibition effect was observed in a delayed naming task and a combination task of delayed naming and articulation suppression^33^. With regard to the word-form encoding stage, immediate naming involves the stages of phonological encoding, phonetic encoding, and articulation; delayed naming involves articulation only; while a combination task of delayed naming and articulation suppression involves the processes of phonetic encoding and articulation. By comparing these effects, these researchers suggested that the segmental facilitation effects were localized at the stage of phonological encoding, whereas the segmental inhibition effects were localized at the stage of phonetic encoding. Zhang and Damian reported a phonemic overlap effect in the time interval between 500–600 ms, which falls within the time window of phonetic encoding. Meanwhile, a weakly inhibitive effect was correspondingly observed^34^. Combining the behavioral and ERP data of previous research with that of the present study, we thus speculate that the facilitative effects in the present study probably arose at the stage of phonological encoding, while the weakly inhibitive effects in the masked priming task probably arose at the stage of phonetic encoding. Obviously, these assumptions need to be examined further.

The differences between the conditions were much larger in the ERP onsets than in the naming latencies. The naming latencies were the time lengths of picture naming before articulation, which include conceptual preparation, lexical selection, and word-form encoding. The difference in naming latencies reflected the overall difference between the related and unrelated conditions in picture naming. By contrast, ERP measurements can provide fine-grained temporal courses of each stage involved in picture naming. Therefore, the comparison between the related and the unrelated conditions from ERP data reflected the differences at specific stages in distinct time intervals (i.e., word-form encoding). From conceptual preparation to word-form encoding, there may exist a cancellation among all stages included in picture naming, which would manifest as a larger difference at a specific stage but a smaller difference overall.

There are two points worthy of mention here. First, in the analysis of ERP data, we observed that the starting point of the syllabic effect was earlier than of the body effect, and the body effect was earlier than the rhyme effect. However, as the time intervals of the three effects were partially overlapped, one possibility we admit worthy of consideration is that the earlier emergence might due to the comparison of a larger effect with a smaller one, even if they occurred quite simultaneously. In this study, we adopted two methods to measure the starting points of those effects. Results from both methods provided consistent evidence, whereas the question still remains open that how to compare the starting point of a larger effect with a smaller effect when they had overlapped time windows. Second, following previous studies, we conducted repeated ANOVA analysis to investigate the modulations of distractor types on ERPs. The repeated ANOVA only includes subjects as random factor and ignores by-item random effects. We think that the use of ANOVA in ERP study might due to the aim to obtain a reasonable signal-to-noise ratio, as EEG signals are sensitive to artefacts. Although for naming latency analysis we found that models including or excluding by-item random effects did not differ significantly (*p* = 0.80), it should be acknowledged that this limited the generalization of the results to other items and future studies might explore the influence of by-item random effects directly.

To summarize, the current study first provides a fine-grained time course of syllabic and segmental encoding in Chinese spoken word naming. Syllables are retrieved before segments, and segments and their order are retrieved incrementally from left to right in the production of Chinese spoken words. Our findings provide evidence for the hypothesis that the phonological units used in spoken word production vary across languages.

## Methods

### Participants

Twenty undergraduate and graduate students (4 males and 16 females; average age 22.05 years; range 18–28 years; all right-handed) participated in the experiment. These students were recruited from Beijing Forestry University, China Agriculture University, and Beijing Science and Technology University. All were native Mandarin Chinese speakers with normal or corrected-to-normal vision. They were each paid 80 yuan (approximately 12 USD) for their participation. Informed consent was obtained from each participant prior to the experiment. This study followed the ethical procedures for the protection of human participants in research and was approved by the ethics committee of the Institute of Psychology, Chinese Academy of Sciences.

### Materials

Eighteen black-on-white line drawings of common objects with monosyllabic names were selected as targets from Zhang and Yang’s picture-naming database of Chinese^52^. All of the target names had a CVN syllabic structure (C represents consonants, V represents vowels, and N represents nasal sounds), e.g., 镜 (/jing4/). Each target was paired with 4 single-character distractors whose names shared either an atonal syllable (same segmental composition but different tone, e.g., 景, /jing3/, hereafter referred to as the syllable-related condition); the initial 2 phonemes (same consonant and vowel but different tone, e.g., 姐, /jie3/, hereafter referred to as the body-related condition); the final 2 phonemes (same vowel and nasal sound but different tone, e.g., 倾, /qing1/, hereafter referred to as the rhyme-related condition); or no phonological overlap (e.g., 捞, /lao1/; hereafter referred to as the unrelated condition) with the target (see Fig. 1a). Semantic or orthographic overlap between distractors and targets was avoided. Frequencies of monosyllabic words (Modern Chinese Frequency Dictionary, 1986) and stroke numbers were matched across conditions (frequency: *F*_(3, 68)_ = 0.001, *p* = 1; stroke number: *F*_(3,68)_ = 0.1, *p* = 0.96). All stimuli are presented as a supplementary file in the Supplementary Material section.

In order to counterbalance the trials of the phonologically related and unrelated conditions, a set of 9 pictures was used as filler and paired with 4 unrelated single characters. Consequently, half of the distractor-target pairs were phonologically related, and half were phonologically unrelated. In addition, there were 6 practice trials in which 3 pictures were paired with one unrelated single character; each pair was presented twice.

### Design

The experiment adopted a factor of distractor type (syllable-related, body-related, rhyme-related, and unrelated) as a within-subject design. Due to the limitations of available items, we manipulated the repetition exactly the same way as J.-Y. Chen *et al*.^2^ and Wang *et al*.^21^. Each block included six practice trials, 72 target trials (18 targets paired with four different kinds of distractors), and 36 filler trials (nine fillers paired with four unrelated distractors), resulting in 114 trials. Each repetition was set in one block, and three repetitions resulted in three blocks and 342 trials in total. The picture order (targets and fillers) within a block was pseudo-randomized in order to prevent targets from repeating within five trials. A new sequence was generated for each participant and each block.

### Apparatus

The experiment was performed using E-Prime Professional Software (version 1.1; Psychology Software Tools). Participants were seated in a quiet room approximately 70 cm from a 21-inch CRT computer screen with a refresh rate of 100 Hz. Naming latencies were measured from target onset using a voice-key, connected to the computer via a PST Serial Response Box.

### Procedure

Participants were tested individually in front of a computer in a soundproof room. Participants were first asked to familiarize themselves with the 30 pictures by viewing each for 2,000 ms, with the names printed below each picture. Following the learning phase, participants underwent a picture-naming test, in which the names were not presented with the pictures. When all of pictures had been named correctly, the experimental blocks were administered.

Each trial involved the following sequence: a fixation ‘+’ presented at the center of the screen for 500 ms, followed by a blank screen for 300 ms, followed by the presentation of a target picture on which a distractor was superposed. Participants were instructed to ignore the distractors and name the pictures as accurately and as quickly as possible. An inter-trial interval of 1,000 ms concluded each trial. Naming latencies were measured from the onset of the picture. The experiment took a total of approximately 25 minutes, and there was a short break between the two blocks.

### EEG recordings and analysis

EEGs were recorded using a Neuroscan amplifier (Neuroscan SynAmps) through 66 electrodes located at the standard 10–20 scalp sites secured in an elastic cap (Electro Cap International). The vertical electro-oculogram (VEOG) was monitored with electrodes affixed above and below the left eye. The horizontal EOG (HEOG) was recorded with a bipolar montage using two electrodes placed on the right and left external canthi. The left mastoid electrode served as a reference. Impedances of all electrodes were kept below 5 kΩ. Electrophysiological signals were amplified with a band-pass filter of 0.05 and 70 Hz and digitized continuously at a rate of 500 Hz.

Neuroscan 4.3 software was used to analyze the ERP data. Waveforms of all single trials were first visually inspected and epochs contaminated by eye movements, electrode drifting, amplifier blocking, and EMG artifacts or other noise were rejected. The EEG data were re-referenced off-line to the average of both mastoids and filtered off-line using a 0.1-Hz high-pass filter and a 30-Hz low-pass filter. The EEG data were segmented from 100 ms before to 500 ms after the onset of the pictures, with baseline corrections ranging from −100 to 0 ms preceding picture onset. Epochs with amplitudes exceeding ±100 μV were rejected. The remaining epochs were considered in ERP analyses. For each distractor type, the mean amplitudes of the ERP waveforms were analyzed. To provide a more detailed depiction of the effect distribution on the scalp, 9 regions of interest (ROIs) were selected along the sagittal and coronal axes: the left-anterior (F3, F5, FC3), mid-anterior (Fz), right-anterior (F4, F6, FC4), left-central (C3, C5), mid-central (Cz), right-central (C4, C6), left-posterior (P3, P5, PO3), mid-posterior (Pz), and right-posterior (P4, P6, PO4). The ROIs were in accordance, or had considerable overlap, with those of previous studies^25,26,53^.

## Supplementary information

Materials

## Data Availability

The data of the current study are available from the corresponding author upon reasonable request.

## References

[1] Levelt WJM, Roelofs A, Meyer AS (1999). A theory of lexical access in speech production. Behav Brain Sci.

[2] Chen J-Y, Lin W-C, Ferrand L (2003). Masked priming of the syllable in Mandarin Chinese speech production. *Chinese*. Journal of Psychology.

[3] O’Seaghdha PG, Chen JY, Chen TM (2010). Proximate units in word production: Phonological encoding begins with syllables in Mandarin Chinese but with segments in English. Cognition.

[4] Schiller NO (1998). The effect of visually masked syllable primes on the naming latencies of words and pictures. J Mem Lang.

[5] Schiller NO (1999). Masked syllable priming of English nouns. Brain Lang.

[6] Schiller NO (2000). Single word production in English: the role of subsyllabic units during phonological encoding. J Exp Psychol Learn Mem Cogn.

[7] Verdonschot RG (2011). The Functional Unit of Japanese Word Naming: Evidence from Masked Priming. J Exp Psychol Learn Mem Cogn.

[8] Meyer AS (1991). The time course of phonological encoding in language production: Phonological encoding inside a syllable. J Mem Lang.

[9] Meyer AS, Schriefers H (1991). Phonological facilitation in picture-word interference experiments: Effects of stimulus onset asynchrony and types of interfering stimuli. J Exp Psychol Learn Mem Cogn.

[10] Roelofs A (1999). Phonological Segments and Features as Planning Units in Speech Production. Lang Cogn Processes.

[11] Schiller NO (2004). The onset effect in word naming. J Mem Lang.

[12] Damian MF, Dumay N (2007). Time pressure and phonological advance planning in spoken production. J Mem Lang.

[13] Collins AF, Ellis AW (1992). Phonological priming of lexical retrieval in speech production. Br J Psychol.

[14] Damian MF, Dumay N (2009). Exploring phonological encoding through repeated segments. Lang Cogn Proc.

[15] Timmer K, Ganushchak LY, Mitlina Y, Schiller NO (2014). Trial by trial: selecting first or second language phonology of a visually masked word. Lang Cogn Neurosci.

[16] Jouravlev, O., Lupker, S. J., & Jared, D. Cross-language phonological activation: evidence from masked onset priming and ERPs. *Brain Lang*, 11–22 (2014). 10.1016/j.bandl.2014.04.003 24814580

[17] Timmer K, Vahidgharavi N, Schiller NO (2012). Reading aloud in Persian: ERP evidence for an early locus of the masked onset priming effect. Brain Lang.

[18] Chen JY, Chen TM, Dell GS (2002). Word-Form Encoding in Mandarin Chinese as Assessed by the Implicit Priming Task. J Mem Lang.

[19] Zhang Q (2008). Phonological Encoding in Monosyllabic and Bisyllabic Mandarin Word Production: Implicit Priming Paradigm Study: Phonological Encoding in Monosyllabic and Bisyllabic Mandarin Word Production: Implicit Priming Paradigm Study. Acta Psychologica Sinica.

[20] You, W., Zhang, Q. & Verdonschot, R. G. Masked Syllable Priming Effects in Word and Picture Naming in Chinese. *PLoS One*, **7**(10) (2012). 10.1371/journal.pone.0046595 PMC3466322 23056360

[21] Wang, J., Wong, A. W., Wang, S., & Chen, H. Primary phonological planning units in spoken word production are language-specific: Evidence from an ERP study. *Sci Rep*, **7**(1) (2017). 10.1038/s41598-017-06186-z PMC5517664 28724982

[22] Chen JY, O’Seaghdha PG, Chen TM (2016). The primacy of abstract syllables in Chinese word production. J Exp Psychol Learn Mem Cogn.

[23] Chen TM, Chen JY (2013). The syllable as the proximate unit in Mandarin Chinese word production: An intrinsic or accidental property of the production system?. Psychon Bull Rev.

[24] Wong AWK, Chen H-C (2008). Processing segmental and prosodic information in Cantonese word production. J Exp Psychol Learn Mem Cogn.

[25] Qu Q, Damian MF, Kazanina N (2012). Sound-sized segments are significant for Mandarin speakers. P Natl Acad Sci USA.

[26] Yu M, Mo C, Mo L (2014). The role of phoneme in mandarin Chinese production: Evidence from ERPs. PLoS One.

[27] Yu, M., Mo, C., Li, Y., & Mo, L. Distinct representations of syllables and phonemes in Chinese production: Evidence from fMRI adaptation. *Neuropsychologia*, 253–259 (2015). 10.1016/j.neuropsychologia.2015.08.027 26334943

[28] O’Seaghdha PG (2015). Across the great divide: Proximate units at the lexical-phonological interface. Jpn Psychol Res.

[29] Roelofs A (2015). Modeling of phonological encoding in spoken word production: From Germanic languages to Mandarin Chinese and Japanese. Jpn Psychol Res.

[30] Wang J, Wong AW, Chen H (2018). Time course of syllabic and sub-syllabic processing in Mandarin word production: Evidence from the picture-word interference paradigm. Psychon Bull Rev.

[31] Laganaro M, Valente A, Perret C (2012). Time course of word production in fast and slow speakers: a high density ERP topographic study. NeuroImage.

[32] Roelofs A, Shitova N (2017). Importance of response time in assessing the cerebral dynamics of spoken word production: Comment on Munding *et al*. (2016). Lang Cogn Neurosci.

[33] Yue Y, Zhang Q (2015). Syllable and Segments Effects in Mandarin Chinese Spoken Word Production (in Chinese). Acta Psychologica Sinica.

[34] Zhang, Q., & Damian, M. F. Syllables constitute proximate units for Mandarin speakers: Electrophysiological evidence from a masked priming task. *Psychophysiology*, e13317 (2019). 10.1111/psyp.13317 30657602

[35] Bates, D. Fitting linear mixed models in R. R News, 5(1), Article 5. Retrieved from, http://ftp.cs.pu.edu.tw/network/CRAN/doc/Rnews/Rnews_2005-1.pdf (2005).

[36] Baayen RH, Davidson DJ, Bates DM (2008). Mixed-effects modeling with crossed 576 random effects for subjects and items. J. Mem. Lang..

[37] Barr DJ, Levy R, Scheepers C, Tily HJ (2013). Random effects structure for confirmatory hypothesis testing: Keep it maximal. J. Mem. Lang..

[38] R Development Core Team R: A language and environment for statistical computing. (version 2.8.1). [Software]. (Vienna, Austria: R Foundation for Statistical Computing, http://www.R-project.org) (2009).

[39] Guthrie D, Buchwald JS (1991). Significance testing of difference potentials. Psychophysiology.

[40] Miller J, Patterson T, Ulrich R (1998). Jackknife-based method for measuring LRP onset latency differences. Psychophysiology.

[41] Indefrey P, Levelt WJM (2004). The spatial and temporal signatures of word production components. Cognition.

[42] Indefrey P (2011). The spatial and temporal signatures of word production components: A critical update. Front Psychol.

[43] Wong, A. W., Wang, J., Ng, T., & Chen, H. Syllabic encoding during overt speech production in Cantonese: Evidence from temporal brain responses. *Brain Res*, 101–109 (2016). 10.1016/j.brainres.2016.07.032 27450928

[44] Zhu X, Damian F, Zhang Q (2015). Seriality of semantic and phonological processes during overt speech in Mandarin as revealed by event-related brain potentials. Brain Lang.

[45] Dell’Acqua, R. *et al*. ERP evidence for ultra-fast semantic processing in the picture-word interference paradigm. *Front Psychol*, 177–177 (2010). 10.3389/fpsyg.2010.00177 PMC3153787 21833238

[46] Wheeldon L, Levelt WJ (1995). Monitoring the time course of phonological encoding. J Mem Lang.

[47] Van Turennout M, Hagoort P, Brown CM (1998). Brain Activity During Speaking: From Syntax to Phonology in 40 Milliseconds. Science.

[48] Levelt WJM (1999). Models of word production. Trends in Cognitive Sciences.

[49] Wong AW, Chen H (2009). What are effective phonological units in Cantonese spoken word planning. Psychon Bull Rev.

[50] Wong AWK, Chen H-C (2015). Processing segmental and prosodic information in spoken word planning: Further evidence from Cantonese Chinese. Jpn Psychol Res.

[51] Finkbeiner M, Caramazza A (2006). Now you see it, now you don’t: On turning semantic interference into facilitation in a Stroop-like task. Cortex.

[52] Zhang Q, Yang Y (2003). The determiners of picture-naming latency (in Chinese). Acta Psychologica Sinica.

[53] Zhu, X., Damian, M. F., & Zhang, Q. Seriality of semantic and phonological processes during overt speech in Mandarin as revealed by event-related brain potentials. *Brain Lang*, 16–25 (2015). 10.1016/j.bandl.2015.03.007 25880902

