# Supplementary material for: Syllables are Retrieved before Segments in the Spoken Production of Mandarin Chinese: An ERP Study
Source: Sci Rep. 2019 Aug 13;9:11773. doi: 10.1038/s41598-019-48033-3 (PMC6692332; doi:10.1038/s41598-019-48033-3)
Supplement: Supplementary file 1 — Materials [file 41598_2019_48033_MOESM1_ESM.pdf]

Syllables are Retrieved before Segments in the Spoken Production of Mandarin Chinese: An ERP Study

Chen Feng[2], Yuan Yue[2], Qingfang Zhang[1*]

[1] *Department of Psychology, Renmin University of China, Beijing, 100872, China*

[2] *Key Laboratory of Behavioral Science, Institute of Psychology, Chinese Academy of Sciences, Beijing, 100101, China*

*Correspondence to qingfang.zhang@ruc.edu.cn

target	pronounciation	meaning	syllable distractor	pronounciation	meaning	initian segment-distractor	pronounciation	meaning	final segment-distractor	pronounciation	meaning	unrelated	pronounciation	meaning
盘	pan2	plate	判	pan4	judge	炮	pao4	cannon	惨	can3	miserable	给	gei3	give
盆	pen2	pot	喷	pen1	spray	配	pei4	assign	笨	ben4	stuipid	招	zhao1	call
瓶	ping2	bottle	娉	ping1	grace	瞥	pie3	glimpse	幸	xing4	luck	稻	dao4	rice
蛋	dan4	egg	耽	dan1	delay	逮	dai3	catch	蓝	lan2	blue	丘	qiu1	mound
盾	dun4	shield	吨	dun1	ton	堆	dui1	pile	晕	yun1	dizzy	埋	mai12	bury
糖	tang2	sugar	躺	tang3	lie	套	tao4	set	放	fang4	place	妞	niu1	girl
铃	ling2	bell	另	ling4	another	烈	lie4	fierce	鼎	ding3	cauldron	泡	pao4	bubble
蝉	chan2	cicada	颤	chan4	shiver	拆	chai1	unknit	贪	tan1	greedy	穗	sui4	spike
唇	chun2	lip	春	chun1	spring	炊	chui1	cooking	婚	hun1	marriage	悲	bei1	sorrow
山	shan1	hill	善	shan4	kind	晒	shai4	bathe	感	gan3	feel	脆	cui4	fragile
琴	qin2	piano	亲	qin1	intimate	恰	qia4	exactly	敏	min3	sensitive	腰	yao1	waist
信	xin4	letter	欣	xin1	glac	携	xie2	bing	音	yin1	sound	豪	hao2	proud
星	xing1	star	悻	xing4	sad	夏	xia4	summer	亭	ting2	pavilion	烤	kao3	bake
镜	jing4	mirror	景	jing3	scene	姐	jie3	sister	倾	qing1	pour	捞	lao1	drag
笋	sun3	bamboo shoot	孙	sun1	grandson	碎	sui4	broken	吞	tun1	swallow	费	fei4	fee
羊	yang2	sheep	央	yang1	center	药	yao4	medicine	让	rang4	avoid	磊	lei3	build
碗	wan3	bowl	完	wan2	finish	外	wai4	outside	刊	kan1	journal	内	nei4	inside
烟	yan1	smoke	演	yan3	play	摇	yao2	wave	燃	ran2	burn	袖	xiu4	sleeve
